# Selection Tool for Foodborne Norovirus Outbreaks

**DOI:** 10.3201/eid1501.080673

**Published:** 2009-01

**Authors:** Linda P.B. Verhoef, Annelies Kroneman, Yvonne van Duynhoven, Hendriek Boshuizen, Wilfrid van Pelt, Marion Koopmans, on behalf of the Foodborne Viruses in Europe Network

**Affiliations:** National Institute for Public Health and the Environment, Bilthoven, the Netherlands

**Keywords:** Norovirus, surveillance, foodborne, epidemiology, virology, gastroenteritis, communicable diseases, outbreaks, prevention and control, research

## Abstract

Detection of pathogens in the food chain is limited mainly to bacteria, and the globalization of the food industry enables international viral foodborne outbreaks to occur. Outbreaks from 2002 through 2006 recorded in a European norovirus surveillance database were investigated for virologic and epidemiologic indicators of food relatedness. The resulting validated multivariate logistic regression model comparing foodborne (n = 224) and person-to-person (n = 654) outbreaks was used to create a practical web-based tool that can be limited to epidemiologic parameters for nongenotyping countries. Non–genogroup-II.4 outbreaks, higher numbers of cases, and outbreaks in restaurants or households characterized (sensitivity = 0.80, specificity = 0.86) foodborne outbreaks and reduced the percentage of outbreaks requiring source-tracing to 31%. The selection tool enabled prospectively focused follow-up. Use of this tool is likely to improve data quality and strain typing in current surveillance systems, which is necessary for identification of potential international foodborne outbreaks.

## Abstract 2

Surveillance data provided a practical tool, which prospectively selects potential food-related norovirus outbreaks.

Globalization of the food industry, centralized production, and the wide geographic distribution of products support the need for increased international surveillance of foodborne viral outbreaks, which may occur in clusters in different countries. Because control of pathogens in the food chain requires hazard analysis critical control points and verification of measures taken, detection of the pathogen is an important step (*1*). However, viral contamination of food is less likely to be recognized than bacterial contamination due to the infrequency of testing for viruses (*2*). Moreover, foods acceptable by bacterial standards are not necessarily safe from viral contamination. For example, norovirus may be present in shellfish and it may still meet the European Union *Escherichia coli* standard for human consumption (*3*). Consequently, foodborne viral infections are common, despite successful measures to reduce bacterial contamination. Recognition of foodborne viral outbreaks with international consequences would benefit from a linked and consistent reporting network among countries.

The challenge for surveillance systems is to obtain a complete dataset for the reported outbreaks (*4*). The European Food Safety Authority (EFSA) recognized this challenge and began developing guidelines for an international reporting system for foodborne outbreaks caused by bacteria, viruses, or parasites (working group of foodborne outbreak surveillance, www.efsa.europa.eu). The need for a better surveillance system has also been recognized by the Foodborne Viruses in Europe (FBVE) network, which has conducted virus-specific surveillance of gastroenteritis outbreaks since 1999 (*5*). Although the name FBVE suggests a foodborne focus, the network actually investigates outbreaks from all modes of transmission to obtain a comprehensive overview of viral activity in the community. A total of 13 countries are participating in the FBVE surveillance network, 11 of which are capable of collecting integrated epidemiologic and virologic surveillance data (*6*).

Because of the etiologic dominance of viruses, the network’s primary focus is on norovirus infections (*7*) that have been more frequently reported in recent years after emergence of novel variant strains in the population (*8*,*9*). Kroneman et al. described strengths and limitations of the FBVE data collection (*6*) but stated that outbreak reports need to be interpreted with caution; the number and content of these reports may vary considerably among countries because surveillance databases may be different. Most of these reports link outbreaks to person-to-person transmission; international interventions and follow-up are rare. In ≈40% of the outbreaks, no suspected mode of transmission was reported. Therefore, epidemiologic or virologic criteria should be used during the early stages of an outbreak investigation to determine whether foodborne sources should be considered. Given that surveillance systems are overwhelmed during norovirus peak seasons, use of these criteria would assist in focusing follow-up activities.

Our objective was to retrospectively derive, from surveillance data, a predictive model that could serve prospectively in the selection of norovirus outbreaks potentially related to food. Such a tool could be used to warn food safety authorities (FSAs) earlier, to improve the quality of outbreak report data, and to provide better estimates of the effects of viral foodborne disease. Our study demonstrates the added value of a reporting system amalgamated across countries; the FBVE dataset can form the basis of this tool, which may be a first step towards detection of diffuse outbreaks.

## Methods

### Categorizing Surveillance Systems

Because surveillance systems are known to vary in terms of design, effectiveness, and priorities (*6*,*9*–*11*), a structured telephone survey/questionnaire (available from the authors) was conducted among FBVE participants to categorize national surveillance systems of the involved countries. If a participating country reported that their national system labeled outbreaks “person-to-person” as a diagnosis of exclusion, the data were excluded to avoid potential misclassification of foodborne outbreaks and consequent dilution of differentiating parameters.

### Dataset

Combined epidemiologic and virologic outbreak reports from countries capable of detecting foodborne outbreaks derived from the FBVE network were collected in a protected web-based database on the basis of a structured questionnaire (www.fbve.nl/attachments/questionnaire.pdf). Collection of sequence results focused on region A of the genome (www.rivm.nl/bnwww), but allowed other entries (regions B, C, and D) because of the lack of standardization between cooperating laboratories (*12*). Norovirus outbreaks were selected from this database if they fulfilled the following minimum dataset: date of onset from January 1, 2002, through December 31, 2006; norovirus detected as the only causative agent; and presence of a known norovirus sequence or genotype. The surveillance database from April 2007 was used and accounted for the median reporting lag (*6*) and enabled completion of data entry for outbreaks in 2006.

### Parameters for Evidence

Data items used in the construction of the predictive model were derived from the EFSA draft guidelines (www.efsa.europa.eu), which are being developed to achieve consensus on the minimal variables to be reported for all foodborne outbreaks and on additional variables to be reported for thoroughly-investigated foodborne outbreaks. The list has been amended with data items for foodborne outbreaks as described in comprehensive overviews (*13*,*14*) and with data items required to enable interventions by an FSA (Table 1).

**Table 1:** Consensus list of parameters for optimal reporting of foodborne (viral) outbreaks as defined by expert opinion, and completeness for data collected in the FBVE surveillance database*

Parameters for outbreak data	Variable	Foodborne outbreak data (% missing ), n = 224	Other mode outbreak data (% missing), n = 654
EFSA (confirmed/probable)†			
Type of outbreak: general or household	Yes	224 (0)	654 (0)
No. human cases‡	Yes	217 (3)	651 (0)
No. hospitalizations‡	Yes	78 (65)	295 (55)
No. deaths‡	Yes	66 (70)	195 (70)
Foodstuff implicated	*Yes*	*93 (58)*	NA‡
Causative agent§	Yes	224 (0)	654 (0)
Setting	Yes	224 (0)	654 (0)
Contributory factors	Yes	202 (10)	482 (26)
*Origin of foodstuff*	No	NA	NA
*Strength of evidence food*	Yes	*224 (0)*	NA
EFSA (thoroughly investigated)†			
Reason reporting	No	NA	NA
Laboratory results food	Yes	*202 (10)*	NA
Place food produced	No	NA	NA
Place food consumed/purchased	Descriptive	*106 (52)*	NA
Age-affected persons	Categorical	11 (95)	73 (89)
Gender-affected persons	Yes	27 (88)	106 (84)
Additional information on agent	Yes	224 (0)	653 (0)
Additional parameters in literature			
Attack rate†	Yes	121 (46)	226 (59)
Seasonality	Yes	149 (33)	484 (26)
Duration of the outbreak†	Yes	90 (60)	265 (59)
Epidemic curve/point source	No	202 (10)	496 (24)
Sequence or variant	Yes	224 (0)	654 (0)
Link with other outbreaks	Yes	22 (90)	15 (98)
Additional parameters VWA experts			
Incubation period	Yes	51 (77)	65 (90)
Illness in food handlers and their family	Partially	*202 (10)*	NA
Presence of ill persons in setting	No	NA	NA

*FBVE, Foodborne Viruses in Europe network; EFSA, European Food Safety Authority; NA, not applicable; VWA, Food and Consumer Products Safety Authority. Parameters listed in italics could not be included in univariate analyses. †Not restricted to viral. ‡ A systematic retrospective check of Dutch data showed that variables for no. cases involved were reported to the national institute by regional health services when the outbreak was ongoing, and that these numbers were not updated when the outbreak had finished. The same situation was reported for other countries during the telephone survey. §Inclusion criterion.

### Definitions

Outbreaks were reported when they satisfied the agreed-upon case definition (a cluster of >2 patients within 2 days showing signs of acute gastroenteritis indicative of norovirus) (*5*,*15*). A gastroenteritis outbreak was ascribed to norovirus based on compatible descriptive epidemiology and laboratory confirmation according to agreed upon criteria (*16*). Because norovirus outbreaks typically occur in winter, an off-seasonal period was defined as May through September; a seasonal period was defined as October through April of the following year. An outbreak was considered foodborne when infection was related to consumption of food contaminated during production or preparation. Where there was laboratory evidence of norovirus in food or analytical epidemiologic evidence for a food source through a case-control or cohort study, the outbreak was defined as confirmed foodborne. When descriptive epidemiologic data indicated a link to food, the outbreak was defined as probably foodborne. A random 50% of the total dataset was used as the training sample to build a model that distinguishes modes of transmission. The remaining 50% was used as the validation sample to validate the model. Sensitivity, or true positives, of the model for foodborne outbreaks was the proportion of the number of foodborne outbreaks correctly labeled as foodborne. Specificity, or true negatives, of the model for foodborne outbreaks was the proportion of the number of outbreaks reportedly due to person-to-person transmission that are indeed classified as person-to-person transmission. The receiver operating characteristics (ROC) curve was the graphic representation of the tradeoff between false negatives and false positives for every possible cut-off. The area under curve (AUC) was used to determine how well the predictor (based on several variables) was able to discriminate between groups (1 = perfect, 0.5 = no discrimination). Positive predictive value (PPV) was the proportion of outbreaks that met the model’s foodborne criteria that are correctly labeled as such, indicating efficiency in reducing the workload of FSAs.

### Data Analysis

Selected norovirus outbreaks were divided among 3 groups: confirmed or probable foodborne outbreaks; outbreaks resulting from person-to-person transmission; and outbreaks with an unknown mode of transmission. Data analysis was performed stepwise. First, completeness of data in the FBVE database with respect to the data-items in the consensus list (Table 1) was determined for outbreaks from foodborne and person-to-person transmission. Descriptive data were given for items relevant to foodborne outbreaks but not applicable to, or available for, outbreaks by person-to-person or unknown transmission. This included information concerning the food vehicle, product-handling hygiene, and place of preparation or consumption.

Second, the consensus list of predefined data items was used to compare foodborne and person-to-person outbreaks in the training sample by using logistic regression models. Variables were included in a multivariate model if they were statistically significant with p<0.10 during univariate analyses and if completeness of the variable was sufficient (80%) to result in a valid model. Because a logistic regression model can only be considered valid if the number of parameters is <10% of the number of outbreaks in the smallest group, analyzed variables were included as continuous where possible. The variables remained in the multivariate model if p values were <0.10, while the backward selection procedure was used or if they were found to be confounding factors for other variables in the model (β values changing at least 10%). The optimal cut-off value was determined in the training sample and validated in the validation sample. When the validated model performed well, the β values for the final model were based on the total dataset, i.e., the validation and the training set together.

Third, the final model was used to create a web-based tool to assist public health workers in selecting outbreaks for further investigation when they receive outbreak reports, and to calculate the predicted individual probability of each outbreak with unknown mode of transmission caused by food. Individual probabilities of food relatedness were summarized to estimate the number of foodborne outbreaks in the unknown group and in the total dataset.

## Results

### Categorizing Surveillance Systems

Of the surveillance systems in the 13 participating countries, 11 met the FBVE network’s reporting criterion of linked laboratory and epidemiologic norovirus outbreak data. Of these 11 countries, 9 were included for analysis of parameters differentiating foodborne from person-to-person outbreaks: Denmark, Finland, France, Hungary, Italy, the Netherlands, Slovenia, Spain, and Sweden. As a result of legislation, surveillance systems in 4 of these 9 countries focused on foodborne outbreaks. Six of 9 countries reported at least 1 typed outbreak per million inhabitants per year (intensive surveillance). Surveillance systems were categorized as follows: 1) intensive surveillance with focus on food; 2) intensive surveillance without focus on food; and 3) no intensive surveillance.

### Data Analysis

**Figure 1:**
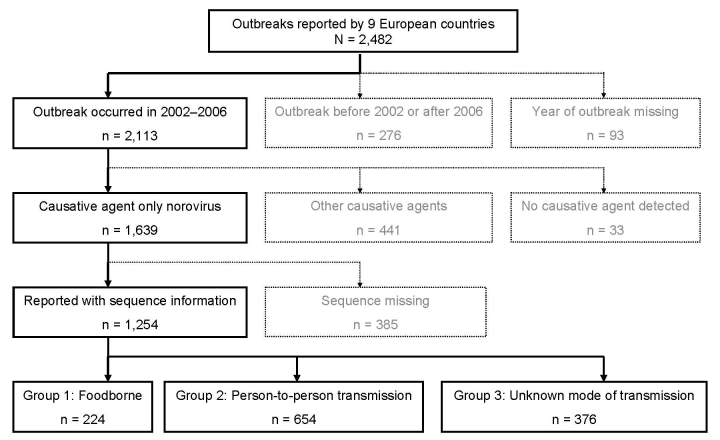
Outbreaks reported to the Foodborne Viruses in Europe network from January 2002 through December 2006, by suspected or confirmed cause and completeness of year and month of the outbreak, sequence information, and mode of transmission. Other causative agents include rotavirus, hepatitis A, and various bacteria.

A total of 1,639 norovirus outbreaks occurring from January 1, 2002, through December 31, 2006, were reported by the countries included. Figure 1 shows the selection of the final dataset comprising 77% (1,254/1,639) of outbreaks; the remaining 23% were excluded due to missing laboratory confirmation of norovirus. Table 1 shows the completeness of analysis-set with respect to the parameters in the consensus list. The level of evidence for food-relatedness was confirmed for 24 (11%) of 224 outbreaks and was probable for 200 (89%) foodborne outbreaks. Thirty food categories were associated with outbreaks, including shellfish, fruit, fancy cakes, buffets, sandwiches, and salads. In 1 foodborne outbreak, poor personal hygiene was mentioned as a contributory factor; an infected food-handler was reported in 16 outbreaks, with 1 cook being involved in 2 outbreaks; and hygiene rather than preparation or consumption of food was mentioned in 2 outbreaks. Completeness of FBVE data with respect to the consensus list of data-items varied between items and between foodborne and person-to-person outbreaks (Table 1). Completeness of data items varied between 2% (link to other outbreaks) and 100% (type of outbreak, setting, causative agent, implicated strain). Data concerning hospitalization, attack rate, epidemic curve, incubation period, and links to other outbreaks were more likely to be reported for foodborne outbreaks. On the other hand, seasonality and contributory factors were more frequently reported for outbreaks with other modes of transmission.

**Figure 2:**
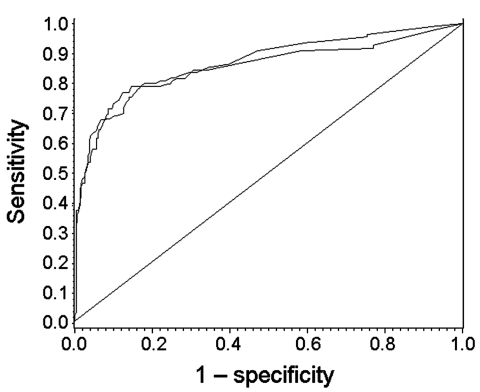
Receiving operator characteristics curves for distinction of foodborne outbreaks from person-to-person outbreaks in the training sample (upper graph, 435/439 records used) and in the validation sample (lower graph, 432/439 records used). The area under curve in the validation sample was 0.90, indicating good performance of the model.

The items in italics in Table 1 could not be included during univariate analyses because they played a role only when foodborne transmission occurred or because data were not requested in the FBVE surveillance system. Risk factors resulting from univariate analyses are presented in Table 2 and show that foodborne outbreaks were found more often in households or restaurants and less often in healthcare settings, involved nongenogroup (GG) II.4 strains relatively more frequently, were more likely to occur during off-seasonal months, and involved more cases when notified compared to outbreaks from person-to-person transmission. Table 2 shows all parameters included in the multivariate analysis that remained in the model during the backward selection procedure. The AUC in the training sample and in the validation sample was 0.92 and 0.90, respectively, indicating the model performs very well in distinguishing foodborne outbreaks from person-to-person transmission (Figure 2). In the validation sample, the optimal cut-off value (the value of the ROC curve closest to the upper left corner) resulted in a sensitivity of 0.72, a specificity of 0.92, and a PPV of 0.64; a follow-up of outbreaks would have focused on 24% of the total number of reported outbreaks. The probability that an outbreak was attributed to food was calculated by using the following final model, based on the complete dataset of 878 records and corrected for characteristics of national surveillance systems:

**Table 2:** Factors (8 of 17) of borderline significance during univariate logistic regression in a random selection of 50% of the dataset for comparison of foodborne outbreaks (group 1) and outbreaks from other modes of transmission (group 2)*

Indicator	Category/measure	Group 1 (n = 112)	Group 2 (n = 327)	Univariate, OR (95% CI)	Univariate adjusted for country, OR (95% CI)	Multivariate adjusted for country, OR (95% CI)
1	General	105	325	Reference	Reference	Reference
	Household	7	2	10.8 (2.2–52.9)	10.1 (1.6–64.3)	0.1 (0.0–1.0)
2	No. cases†	–	–	1.1 (1.0–1.1)	1.0 (0.9–1.1)	1.1 (1.0–1.2)
7	Residence	7	2	Reference	Reference	Reference
	Restaurant‡	36	1	10.3 (0.8–129.4)	13.2 (0.7–234.0)	>999†
	Healthcare institute	27	267	0.0 (0.0–0.1)	0.0 (0.0–0.1)	0.0 (0.0–0.0)
	Daycare	2	15	0.0 (0.0–0.3)	0.1 (0.0–0.9)	0.0 (0.0–0.1)
	Hotel/guest house	9	12	0.2 (0.0–1.3)	0.1 (0.0–1.3)	0.0 (0.0–0.1)
	School	11	9	0.3 (0.1–2.1)	0.3 (0.0–2.7)	0.0 (0.0–0.2)
	Other	20	21	0.3 (0.1–1.5)	0.3 (0.0–2.1)	0.0 (0.0–0.2)
17	Non-GGII.4	55	48	Reference	Reference	Reference
	Genogroup II.4	57	278	0.2 (0.1–0.3)	0.2 (0.1–0.4)	0.4 (0.2–1.0)
*18*	Attack rate*	*–*	*–*	*14.0 (3.6–54.0)*	*6.7 (1.5–34.3)*	*–*
*19*	May–Sep	*20*	*35*	*Reference*	*Reference*	*–*
	Oct–Apr	*47*	*208*	*0.4 (0.2–0.7)*	*0.5 (0.3–1.2)*	
*20*	Duration in hours*	*–*	*–*	*0.9 (0.8–0.9)*	*0.9 (0.8–1.0)*	*–*
*21*	No point source	*60*	*242*	*Reference*	*Reference*	
	Point source	*43*	*3*	*58.8 (17.3–192.7)*	*44.7 (11.8–167.7)*	*–*

*Significant factors were included in multivariate analyses to construct the final model. –, entered as a continuous variable. Parameters in italics could not be included in multivariate analysis because of missing values. OR, odds ratio; CI, confidence interval; GG, genogroup. †A systematic retrospective check of Netherlands data showed that variables for no. of cases involved were reported to the national institute by regional health services when the outbreak was ongoing, and that these numbers were not updated when the outbreak had finished. The same situation was reported for other countries during the telephone survey. ‡The parameter restaurant was set to 0 because the variable was a linear combination of other variables as follows: Restaurant = intercept – household – health care – day care – hotel – school – other >999.

Odds (foodborne = 1)  **=** *P(foodborne=* 1**  ______________ =** *P(foodborne=* 0)

= exp (1.5477 + 0.8065 when in household) + (0.0322 for each involved case when notified) + (3.0999 when in restaurant)  **or** – (1.2963 when in hotel)  **or** – (2.6616 when in hospital)  **or** – (1.9912 when in daycare)  **or** – (0.5289 when in school) + (0.3190 when GGnonII.4) – (1.0270 if intensive surveillance and focus food)  **or** – (2.0540 if intensive surveillance and no focus food)

This final model (sensitivity = 0.80, specificity = 0.86, PPV = 0.65) can be prospectively applied to calculate potential food-relatedness of reported norovirus outbreaks and reduce the number of outbreaks to 31% of all reported outbreaks. The practical web-based tool created with the model can be found in the Technical Appendix. If this tool is used by a nongenotyping country, intensive surveillance can be considered to be at least 2 reported outbreaks (instead of 1 typed outbreak) per 1 million inhabitants per year. For the nongenotyping countries, the unknown genotype in the model resulted in an additional 5 unrecognized foodborne outbreaks coexisting with a slight reduction of sensitivity (0.78), equal specificity (0.86), PPV (0.65), and 30% of the outbreaks requiring follow-up.

Of 376 outbreaks with an unknown mode of transmission, data for 352 (94%) were sufficient to calculate the probability of a foodborne outbreak. Summarizing individual probabilities resulted in 100 (29%) of 352 potential foodborne outbreaks in the unknown group; summarizing probabilities in the total dataset resulted in an estimated 280 (22%) of 1,254 reported outbreaks being possibly foodborne.

## Discussion

We built and validated a model to estimate the likelihood that a norovirus outbreak was related to food. This study was the basis for a practical tool that can prospectively be applied in near real-time in the European setting to identify potential foodborne viral outbreaks in both genotyping and nongenotyping countries. The model can also retrospectively estimate the true contribution of food to norovirus outbreaks in Europe, and may contribute to studies estimating the effects of foodborne gastroenteritis. Moreover, the user-friendly tool may support more consistent reporting and typing of viral outbreaks. Our approach is innovative for norovirus surveillance and provides a new estimate for the proportion of foodborne outbreaks that is higher (22%) than the recognized proportion of foodborne outbreaks in countries that can separate transmission modes (18%). However, this higher estimate is based on reported outbreaks and therefore does not account for underreporting or overreporting.

Using this selection mechanism prospectively for identification of outbreaks requiring detailed follow-up, FSAs can focus on 31% of all reported outbreaks and accept that 1 of 5 foodborne outbreaks will be missed. This finding may appear to lack sensitivity, but at present only a few foodborne outbreaks are investigated sufficiently to provide information on the basis of which FSAs can act, which we will illustrate. In 2007, a year when an unusually high number of norovirus outbreaks were reported, 1 of the 37 Municipal Health Service agencies in the Netherlands reported a total of 28 norovirus outbreaks. Applying this model to reduce the number of outbreaks needing investigation from 28 to 3 is likely to greatly improve the potential for intervention and the quality of surveillance data for foodborne outbreaks. The tool, based on this model, will be implemented and evaluated in the Netherlands in 2009.

As previously identified, restaurants were the most common setting for foodborne outbreaks (*17*,*18*). Our model output, however, also provides a strong first indication that the epidemiology of norovirus outbreaks differs between genotypes because the proportion of non-GGII.4 outbreaks was higher in foodborne outbreaks. Non-GGII.4 outbreaks indicated source contamination, altering the probability of outbreaks being related to food (Technical Appendix). Unfortunately, many countries cannot take advantage of this result because genotyping is not among their routine procedures. For this reason, the practical tool was adjusted so that it can be restricted to epidemiologic parameters only. However, a rapid assay should be developed that discriminates GGII.4 from non-GGII.4, which would enable earlier and more targeted measures by FSAs on a large scale.

The difference identified between GGII.4 and non-GGII.4 is a first step towards identification of international foodborne outbreaks, of which some examples are known (*19*–*21*). Detailed strain type and sequence information may provide the linking conditions for such outbreaks. Unfortunately, analysis of strain types did not give statistically significant results but did suggest the existence of differences, which should be separately investigated. More data are needed about the diversity of noroviruses belonging to rare genotypes to reliably use the data when identifying a probable source of infection. This diversity is illustrated through a recent example. In the spring of 2006, an unusually high number of norovirus outbreaks was reported that involved passengers on cruise ships in European waters (*22*). The finding that several of the outbreaks were caused by a distinct strain of GGII.4 norovirus triggered an outbreak investigation which tested the hypothesis that these outbreaks might have resulted from a common source (*23*). More detailed molecular characterization, outbreak investigations, and use of molecular strain data from surveillance of land-based outbreaks showed that the new variant strain viruses could be found across Europe, thus reflecting a widespread epidemic rather than a common source event. Much less is known about noroviruses belonging to the rare genotypes. For instance, if these viruses behave in an opportunistic fashion, they will circulate in the community without causing outbreaks, going undetected because routine surveillance for sporadic cases is rare (*24*). Until further investigation can show epidemiologic characteristics of rare genotypes, the selection tool using information on setting, genogroup, and number of cases enables quick screening of outbreaks of interest.

Several countries have conducted studies using methods to estimate the proportion of foodborne gastroenteritis (*25*–*27*). These studies have identified foodborne norovirus infections varying from 1/33 inhabitants in the United States to 1/780 in the United Kingdom. Prospective cohort studies are usually the most accurate method of ascribing illness to food, but are costly. Deriving estimates from existing data collections has its weaknesses (*28*), but the data collections available provide a good tradeoff between costs and providing useful information for public health. Numbers based on surveillance data need to be interpreted and extrapolated with caution, as international differences in surveillance systems can introduce bias.

Although our approach is innovative in categorizing norovirus outbreaks in a surveillance system, it is commonly applied in medical research to predict critical diagnostic outcomes (*29*–*31*). A limitation of our data is that selection of outbreaks may have occurred in the database, e.g., if outbreaks were not reported until they had reached a certain size because of (secondary) person-to-person transmission (*32*). Conversely, outbreaks likely to be foodborne may have an origin other than food (*33*), and confirmation of this is rare. In addition, the definition applied for a foodborne outbreak may differ substantially among countries. We reduced the chance of misclassification by using retrospectively applied uniform definitions, and by selecting those countries clearly discriminating transmission modes. During the survey for categorization of surveillance systems, we confirmed that proof of a foodborne outbreak is often difficult to obtain; the transmission mode consequently may remain unknown relatively more frequently than that of person-to-person outbreaks. However, the slight difference between the foodborne proportion of outbreaks among the outbreak with unknown (28%) and known (26%) transmission does not suggest a difference in the estimated and reported proportion.

The European norovirus surveillance system, like most surveillance systems, has to cope with reporting delays and missing values (*4*,*6*,*10*). Because virologic and epidemiologic distinctive parameters were of interest, strict selection criteria were used in this study. Incompleteness in our selection criteria left us with 1,254 (66%) of 1,639 norovirus outbreaks in our analysis dataset. Greater completeness of our analysis dataset may have resulted in an extended model that included a larger variety of indicators as proposed by EFSA, which requested an extensive minimal dataset for foodborne outbreaks from differing causes. However, our model was able to distinguish norovirus foodborne outbreaks with far fewer indicators than those prescribed by EFSA. Because an optimal surveillance system for detection of diffuse foodborne outbreaks is dependent on completeness of a minimal dataset, use of the tool is likely to be a first step towards such a system.

We developed a practical tool that can distinguish food-relatedness of norovirus outbreaks and is likely to improve surveillance data quality. A model that predicts foodborne outbreaks regardless of causative agents and that links conditions for viral outbreaks should be the focus of future studies. The requested minimum dataset for surveillance of foodborne outbreaks with potential for international consequences needs to be clearly defined. The more information needed, the less the compliance; priority should therefore be given to information essential for initiating interventions.

## Supplementary Material

Technical Appendix
